# Study on the Effect of Nanoporous Copper Particle Size on Copper-Based Azide

**DOI:** 10.3390/mi15040462

**Published:** 2024-03-29

**Authors:** Jiabao Wang, Jie Ren, Shuang Li, Mingyu Li, Qingxuan Zeng

**Affiliations:** State Key Laboratory of Explosion Science and Technology, School of Mechatronical Engineering, Beijing Institute of Technology, Beijing 100081, China

**Keywords:** nanoporous copper, particle size control, copper-based azide, micro-initiators

## Abstract

Preparing copper-based azide by in situ reaction is well-suited for MEMS processing technology and holds promising prospects in the field of MEMS micro-initiators. This study involved the preparation of porous copper with particle sizes of approximately 30 nm, 60 nm and 100 nm through powder sintering. These were used as precursors for a gas–solid in situ azide reaction to produce copper-based azide with varying morphologies and compositions. Copper-based azide micro-initiators were designed, and their output performance was evaluated using CL-20 and HNS-IV explosives. Analytical results revealed that the product from the reaction of the 100 nm precursor exhibited a lumpy and uneven structure with a conversion rate of 90.36%. The product from the 60 nm precursor reaction had a dense surface with a conversion rate of 94.56%, while the 30 nm precursor resulted in a needle-like form with a conversion rate of 92.82%. Detonation experiments demonstrated that the copper-based azide micro-initiators prepared with 100 nm of a porous copper precursor exhibited unstable output performance, requiring a 1.6 mg charge to successfully detonate CL-20 explosives. On the other hand, copper-based azide micro-initiators prepared from 60 nm and 30 nm of porous copper precursors exhibited stable output performance. A charge of 0.8 mg was adequate for reliably and consistently detonating CL-20 and HNS-IV explosives. The reduced particle size of the precursor enhanced the output performance of the copper-based azide micro-initiators, providing increased energy redundancy during detonation and improving overall usage reliability.

## 1. Introduction

In recent years, micro-initiators developed on the basis of Micro-Electro-Mechanical Systems (MEMSs) have gained increasing attention due to their advantages of intelligence, mass production, low cost and high reliability [1]. The concern over environmental pollution caused by heavy metal ions in lead azide has been growing in recent years [2,3,4,5], and with the development of miniaturization in weapon systems, the output capacity of lead azide appears somewhat insufficient, potentially limiting its future applications [6,7].

Copper-based azide has a low critical detonation mass and strong output power and is environmentally non-polluting, making it an ideal substitute for lead azide [8,9,10]. The widespread use of in situ reactions significantly reduces operators’ exposure to sensitive explosives and minimizes the risk of accidental explosions involving copper-based azide. There are two primary types of in situ reactions. One method involves using carbon nanotubes or metal–organic frameworks as carriers for the reaction [11,12,13]. In this method, due to the small particle size of the precursor and the sufficiently large space for the expansion of the reaction products, the reaction depth is usually very deep and the conversion rate is high, with the products being mainly Cu(N_3_)_2_, although with a lower charge density. The other method entails preparing a nanoporous copper precursor, which is then press-molded into chips for in situ reaction [14,15,16,17]. The precursor derived from this approach has a larger particle size (100 nm) and higher loading density. However, it exhibits lower reaction depth and yields a mixture of Cu, Cu(N_3_)_2_, and CuN_3_ as the reaction products, leading to unstable output properties.

In this paper, precursors with various particle sizes were prepared through powder sintering. By controlling the particle size, the contradiction of the density of the charge and the depth of the reaction was balanced, and it enhanced the output performance of the copper-based azide micro-initiators.

## 2. Experimental Section

### 2.1. Materials and Equipment

Analytical-grade chemicals (Cu (NO_3_)_2_·3H_2_O, H_2_C_2_O_4_, Cu (CH_3_COO)_2_, H_3_PO_4_, KOH, CaCl_2_, NaN_3_) were purchased and used as received.

X-ray diffraction (XRD) analysis was performed with a diffractometer (D8 ADVANCE; Bruker; Karlsruhe, Germany) with Cu Ka radiation in the range from 10° to 90°. The microstructure of samples was observed by scanning electron microscopy–energy dispersive spectroscopy (SEM-EDS; ZEISS; Oberkochen, Germany). The FTIR spectra of the samples were collected by an infrared spectrometer (TENSOR 27; Bruker; Karlsruhe, Germany) using KBr pellets. The concentration of N_3_^−1^ was determined by UV-visible spectroscopy using copper nitrate as the complexing agent at a wavelength of 375 nm. The absorbance of [CuN_3_] ^+^ was recorded with a spectrophotometer (Agilent Cary 5000; Agilent; Santa Clara, CA, USA), and the N_3_^−1^ concentrations in the solutions were obtained by BeerLambert Law.

### 2.2. Precursor Synthesis

A total of 0.03 mol of Cu (NO_3_)_2_·3H_2_O was accurately weighed and 60 mL of anhydrous ethanol was accurately measured. The copper nitrate was stirred to dissolve it in the anhydrous ethanol under water bath conditions at 70 degrees Celsius. Three separate solutions of the same composition were prepared. In the second and third solutions, copper acetate was added in amounts equivalent to 7% and 4% Cu (NO_3_)_2_·3H_2_O, respectively, and stirred until fully dissolved. Similarly, 0.03 mol of H_2_C_2_O_4_ was weighed and completely dissolved in 60 mL of absolute ethanol under 70 °C water bath conditions, resulting in three identical solutions. Under vigorous stirring using a magnetic stirrer, the oxalic acid solution was rapidly poured into the copper nitrate solution, causing the formation of a light blue flocculent precipitate. The mixture was stirred for an additional 30 min before being stopped. The solvent was evaporated under 70 °C water bath conditions, followed by transfer to a blast drying oven for overnight drying. The resulting material was then ground to obtain a light blue powder. Under nitrogen protection, the material was sintered at a high temperature of 350 °C for 30 min, cooled to room temperature and subsequently retrieved as a nanoporous copper (NPC) precursor.

### 2.3. In Situ Reaction Process

**Figure 1:**
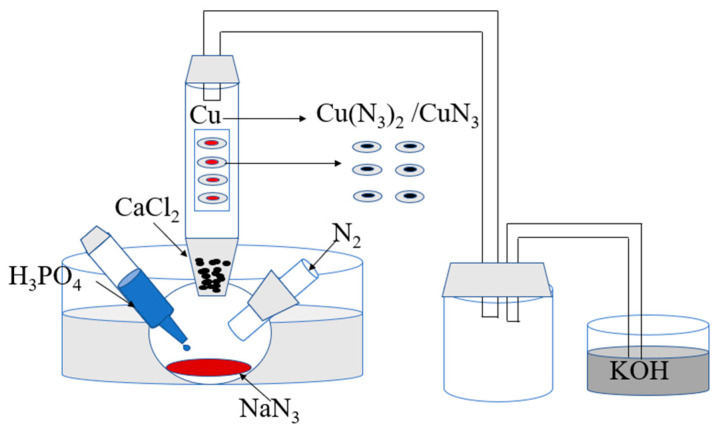
Schematic illustration of in situ reaction process.

The schematic procedure of the in situ reaction is presented in Figure 1. The NPC was pressed into polycarbonate chips. In a typical reaction, gaseous HN_3_ was generated in a three-neck round-bottom flask by heating a mixture of NaN_3_ (2.0 g) with excess H_3_PO_4_ (10.0 mL) at about 50 °C and then introducing calcium chloride (CaCl_2_) driers to remove the moisture. The gaseous HN_3_ could be tested at the top of the glass reactor by the wet iron nitrate (30%) test paper changing to black/red. After the glass reactor was filled with gaseous HN_3_, NPC chips were placed into the glass reactor. All NPC chips were orderly and separately deposited in the reactor for maximum surface area exposure to the gaseous HN_3_. After reaction (24 h), a slow flow of N_2_ (10–15 mL⋅min^−1^) was introduced for 15 min to eliminate residual HN_3_. In addition, the top of the glass reactor was connected to buffer absorption equipment with saturated KOH to filtrate unreacted HN_3_. The azide reaction was handled in a well-ventilated hood.

### 2.4. Initiation Test of Copper-Based Azide Micro-Initiators

A micro-initiation device was designed, it is usually composed of a base plate with a Ni-Cr bridge wire and the available copper-based azide chip, titanium flyer and barrel. The thicknesses of the titanium flyer and barrel were 0.02 mm and 0.50 mm, respectively, and the barrel had the same diameter with a copper-based azide micro-charge. The CL-20 explosive used in the experiment had a column shape of Φ1.5 × 1.0 mm with a density of 1.55 g⋅cm^−3^ [18], and the bare HNS-IV explosive used in the experiment had a column shape of Φ3.0 × 3.0 mm with a density of 1.56 g⋅cm^−3^. Once ignited by an Ni-Cr bridge wire, the copper-based azide could rapidly convert from deflagration to detonation and consequently generate a powerful shock wave to shear a titanium flyer, and then the flyer was accelerated in the barrel. Finally, the CL-20 explosive and HNS-IV explosive were initiated by the flyer with large kinetic energy.

## 3. Results and Discussion

### 3.1. Nanoporous Copper

**Figure 2:**
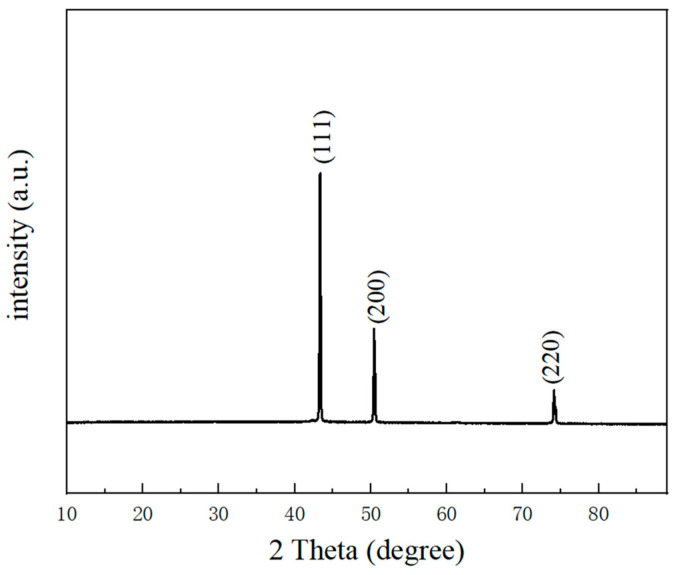
XRD of nanoporous copper.

The morphology and structure of the fabricated NPC were studied by XRD and SEM. As shown in Figure 2, the sintered product was copper with no other impurities, and the peaks at 43.30°, 50.43° and 74.13° corresponded to the reflections of the (111), (200) and (220) crystalline planes of copper (JCPDS No. 04-0836).

**Figure 3:**
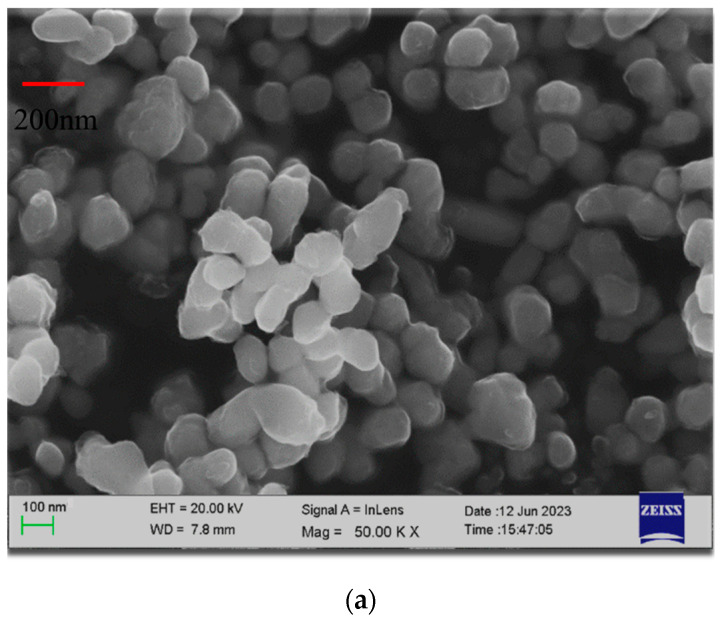

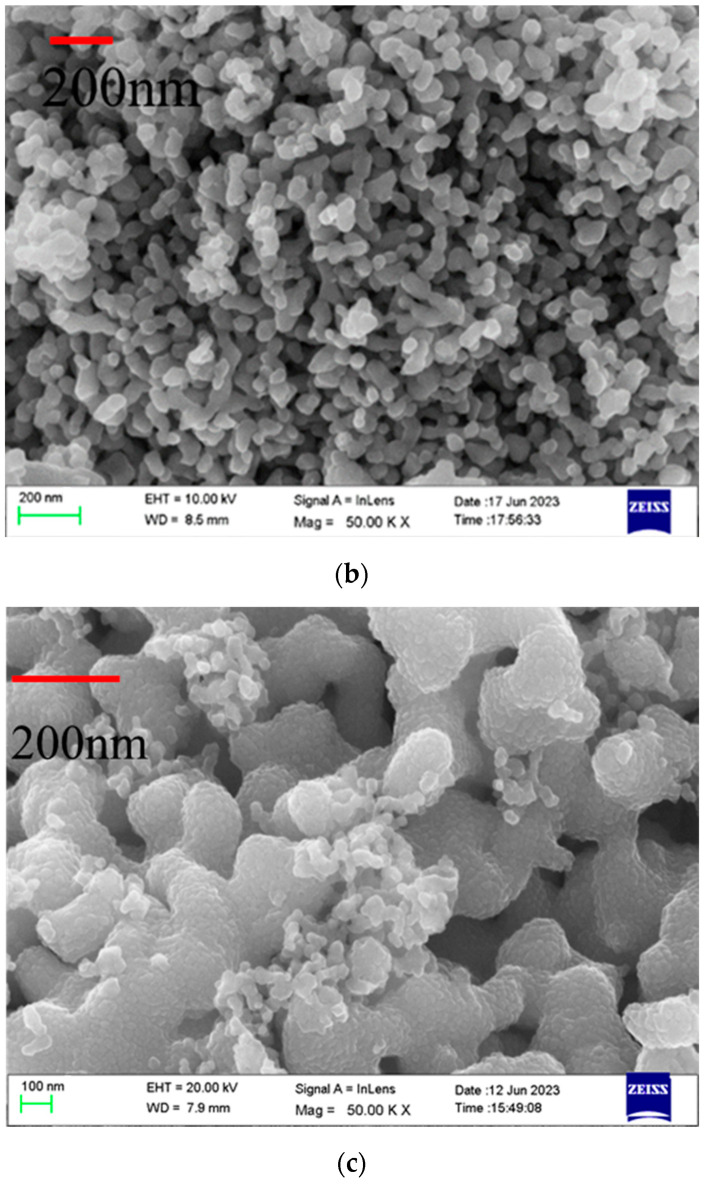
SEM images of nanoporous copper. (**a**) without copper acetate, (**b**) 7% copper acetate, (**c**) 4% copper acetate.

Figure 3a illustrates the preparation process of the precursor without the addition of copper acetate, referred to as Sample 1; Figure 3b portrays the preparation process of the precursor with the addition of 7% copper acetate, denoted as Sample 2; and Figure 3c showcases the preparation process of the precursor with the addition of 4% copper acetate, designated as Sample 3. Scanning electron microscopy revealed that the precursors obtained from the preparation exhibited a uniform size distribution with an ellipsoidal shape. The particle size of Sample 1 was approximately 100 nm, Sample 2 was around 60 nm and Sample 3 was about 30 nm. This indicates that the inclusion of copper acetate in a precursor preparation can decrease the particle size of nanoporous copper and the particle size of the nanoporous copper can be controlled by adjusting the amount of copper acetate added.

### 3.2. Copper-Based Azide

#### 3.2.1. Morphological Analysis of Copper-Based Azide

**Figure 4:**
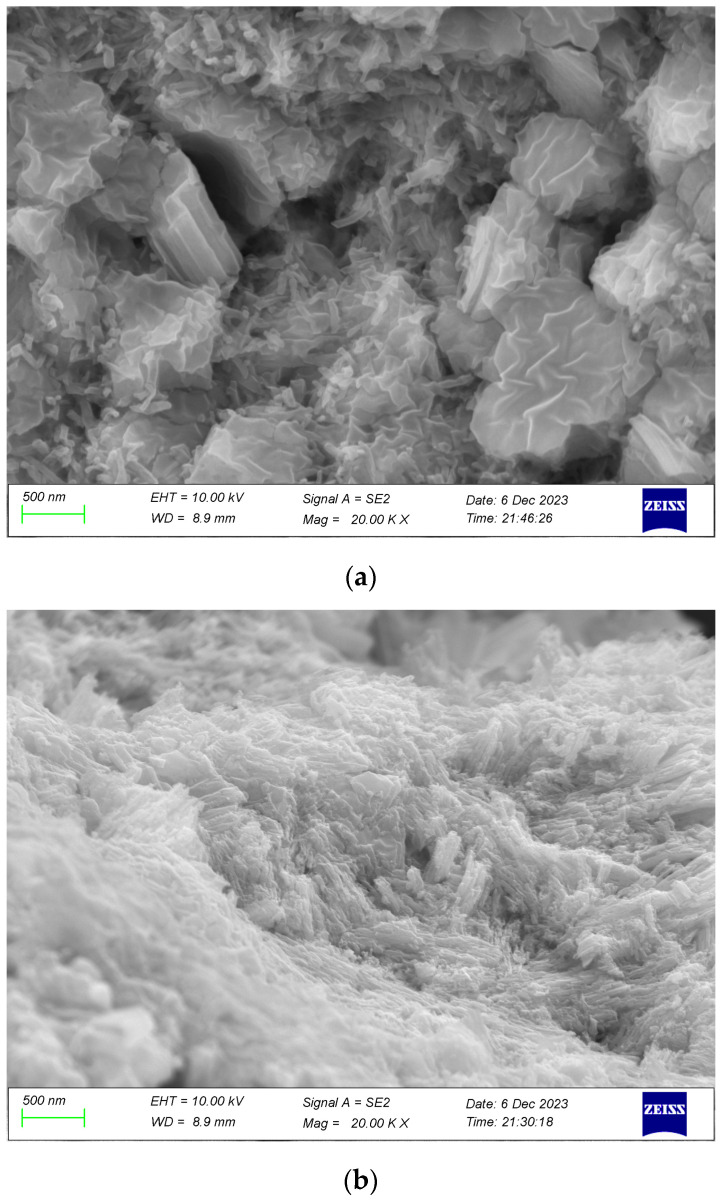

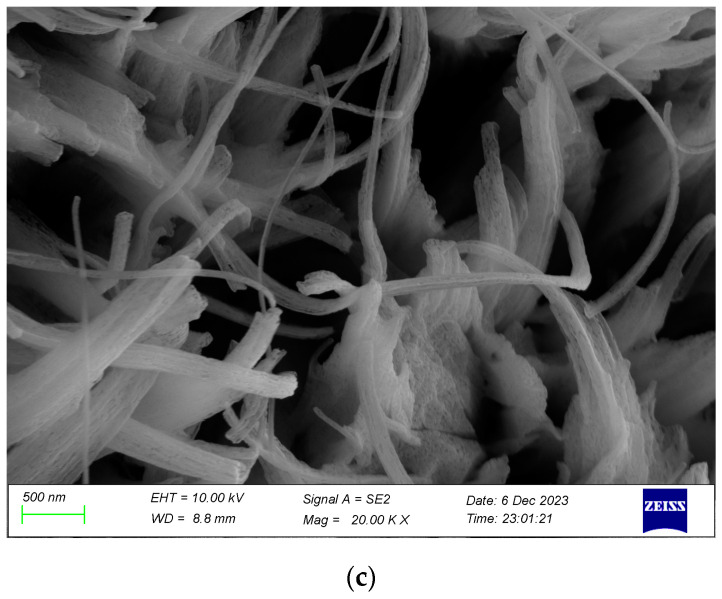
SEM images of copper azide: (**a**) 100 nm of NPC, (**b**) 60 nm of NPC and (**c**) 30 nm of NPC.

The SEM images in Figure 4 illustrate the varied morphologies of copper-based azide observed through scanning electron microscopy, with Figure 4a–c corresponding to Samples 1–3, denoted as Samples a, b and c, respectively. The distinct morphologies of the reaction products are attributed to the differing particle sizes of the precursors. The gas–solid reaction between the nanoporous copper precursors and HN_3_ gas progressed through stages of product island formation, product layer growth and solid-phase diffusion, along with Ostwald ripening processes [19]. Initially, the gas–solid reaction was initiated on the reactant surface, leading to the gradual formation of needle-like or rod-shaped product islands. These islands exhibited directional growth, resulting in an increased aspect ratio. Subsequently, a continuous product layer developed on the reactant surface, representing a material transfer process primarily occurring within a single nanoporous copper precursor. As the product layer thickened, the gas–solid reaction rate diminished and thermodynamic equilibrium drove Ostwald ripening. This process involves individual products coming into contact, facilitating solid-phase material transfer and increasing contact area while decreasing the aspect ratio of the reaction products, ultimately yielding a dense and smooth surface. The experimental findings highlight how precursor particle size influences final product morphology, with varying degrees of Ostwald ripening based on precursor particle sizes. For instance, Sample a corresponds to a nanoporous copper precursor with a particle size of approximately 100 nm, impeding HN_3_ gas diffusion channels early in the maturation process due to material expansion, resulting in a lower degree of maturation. In contrast, the nanoporous copper precursor in Sample c, with a particle size of around 30 nm, experienced rapid consumption during ripening, leading to a lower degree of ripening and prominent needle-like product shapes with reduced product density. Sample b corresponds to a precursor particle size of about 60 nm, which will neither block the diffusion channel of HN_3_ nor show precursor insufficiency during a reaction. This ensures a higher degree of Ostwald maturation, a dense and smooth surface of the reaction product and a higher product density.

#### 3.2.2. Composition Analysis of Copper-Based Azide

**Figure 5:**
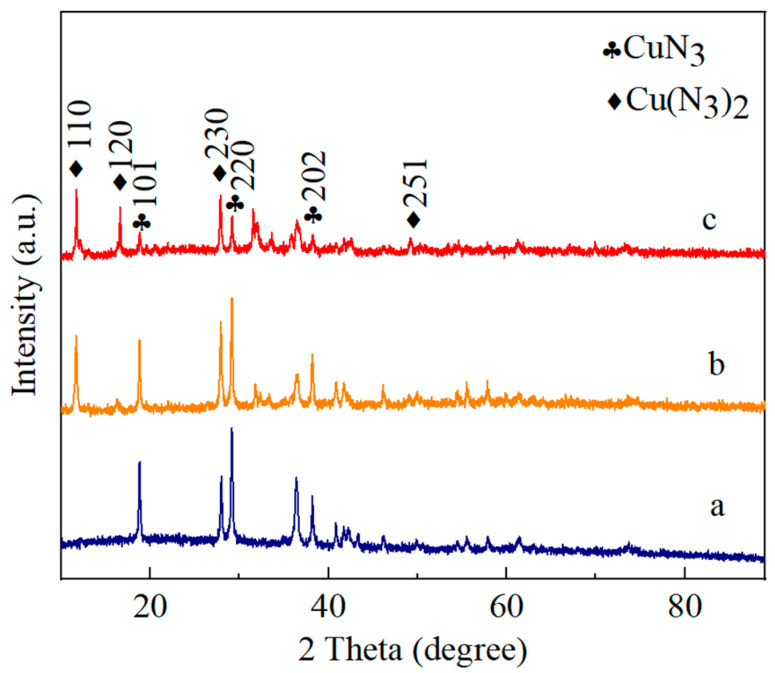
XRD patterns of products with different precursors: (a) 100 nm of NPC, (b) 60 nm of NPC and (c) 30 nm of NPC.

The composition analysis of the copper-based azide was conducted using an X-ray diffractometer, where La Kα rays were utilized within a scanning range of 10° to 90°. The results presented in Figure 5 reveal that the copper azide primarily consisted of CuN_3_ and Cu(N_3_)_2_. The test results indicate that in Sample a, the diffraction peaks were mainly attributed to CuN_3_, with no apparent diffraction peaks of Cu(N_3_)_2_ observed. In Samples b and c, in addition to CuN_3_, distinct diffraction peaks corresponding to the (110) and (230) crystal planes of Cu(N_3_)_2_ were also present. In Sample c, noticeable diffraction peaks for the (120) crystal plane of Cu(N_3_)_2_ were also observed. Alongside the CuN_3_ and Cu(N_3_)_2_, two distinct diffraction peaks were observed at 31.58° and 32.1°. Since copper-based azides are very sensitive, they need to be wetted with water in advance in order to prevent accidental explosion of the copper-based azide during sample preparation and testing; therefore, these peaks may correspond to the hydrolysis products of the copper-based azide. XRD examinations further indicated that the proportion of Cu(N_3_)_2_ in the reaction product increased as the precursor particle size diminished. This suggests that by manipulating the precursor particle size, one can influence the depth of the reaction and regulate the composition of the reaction product. Such control over composition can subsequently govern the output performance of micro-initiators.

**Figure 6:**
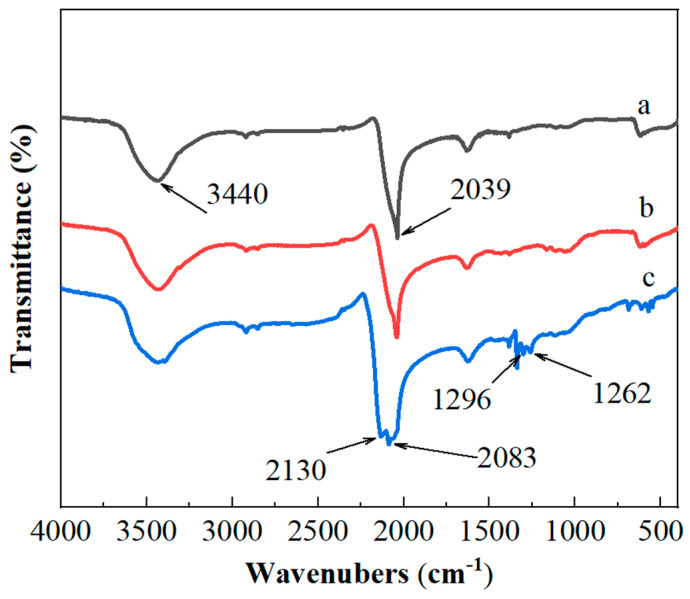
FT-IR spectra of products with different precursors: (a) 100 nm of NPC, (b) 60 nm of NPC and (c) 30 nm of NPC.

The infrared scanning of the reaction products was performed using the KBr pellet method, and the results are shown in Figure 6. The scanning range was set from 4000 to 400 cm^−1^. At 3440 cm^−1^, there is a stretching vibration peak corresponding to the hydroxyl groups. Due to the material’s sensitivity, the sample was moistened during preparation, resulting in water content in the sample. The peak at 2039 cm^−1^ is a characteristic infrared peak of N_3_^−1^. The peaks located at 2083 cm^−1^ and 2130 cm^−1^ represent the asymmetric vibration of N_3_^−1^, while the peaks at 1262 cm^−1^ and 1296 cm^−1^ correspond to the symmetric vibration of N_3_^−1^. These observations indicate the formation of copper-based azide through the reaction.

#### 3.2.3. Compositional Analysis and Bulk Energy Density of Copper-Based Azide

Copper-based azide microcharges synthesized by gas–solid in situ reactions are typically multi-component mixtures. From an energy perspective, the micro-initiation explosive sequence necessitates that the unit volume of copper-based azide microcharges should possess as high of a total chemical energy as possible. This value is dependent on the conversion rates of Cu(N_3_)_2_ and CuN_3_ in the products and the precursor density. It can be calculated based on Equation (1):

(1) $$E_{v}=\frac{\rho }{M}\alpha_{1}\Delta_{f}H_{m,1}^{0}+\frac{\rho }{M}\alpha_{2}\Delta_{f}H_{m,2}^{0}$$

where *ρ* is the loading density of copper powder, g·cm^3^, and in the experiment, the molding density of the samples was about 1.45 g·cm^−3^. *M* is the molar mass, g/mol, and the molar mass of Cu was 63.55 g/mol; $\Delta_{f}H_{m}^{0}$ is the standard molar enthalpy of the generation of the substance, kJ/mol, in which the standard molar enthalpies of generation of Cu, CuN_3_ and Cu(N_3_)_2_ were 0, 281 and 587 kJ/mol [20]; *E_v_* is the energy density per unit volume, J/mm^3^; and *α* denotes the conversion rate. The conversion rates of the CuN_3_ and Cu(N_3_)_2_ were obtained by the copper nitrate method [21], which was tested with the aid of a UV-visible spectrophotometer at a wavelength of 375 nm. When the mass concentration of N_3_^−1^ was in the range of 5–25 mg/L, the relationship equation between absorbance *y* and the N_3_^−1^ concentration (*x*, mg/L) was calculated as (2):

(2) $$y=0.03368x+0.0135,\;R^{2}=0.9999$$

The component masses of Cu(N_3_)_2_ and CuN_3_ in the reaction product satisfy Equation (3):

M = C * V * *N*/*ω* (3)

where *C* is the measured N_3_^−1^ concentration, μg/mL; *V* is the volume of the tested sample, mL; *N* is the dilution factor; and *w* is the mass fraction of N_3_^−1^ in the Cu(N_3_)_2_ and CuN_3_ fractions, corresponding to values of 56.94% and 39.80%, respectively. In order to ensure the accuracy of the test results, five tests were repeated during the experiment to obtain the average value, and the test results of each component of the copper-based azide are shown in Table 1. The volumetric energy density was calculated from the test results.

As shown in Table 1, the conversion rates of the three precursors with different particle sizes reached 90% after the reaction, leaving approximately 10% of unreacted copper precursors. The composition of the final product varied significantly, with Sample a containing 11.32% Cu(N_3_)_2_, Sample b containing 34.31% Cu(N_3_)_2_ and Sample c containing 36.56% Cu(N_3_)_2_. Volumetric energy density calculations indicated that a higher Cu(N_3_)_2_ content in the product resulted in a greater volumetric energy density. The volumetric energy density of Samples b and c was around 30% higher than that of Sample a. This suggests that Products b and c had higher energy for the same charge volume, leading to more reliable output performance.

### 3.3. Initiation Test of Copper-Based Azide Micro-Initiators

**Figure 7:**
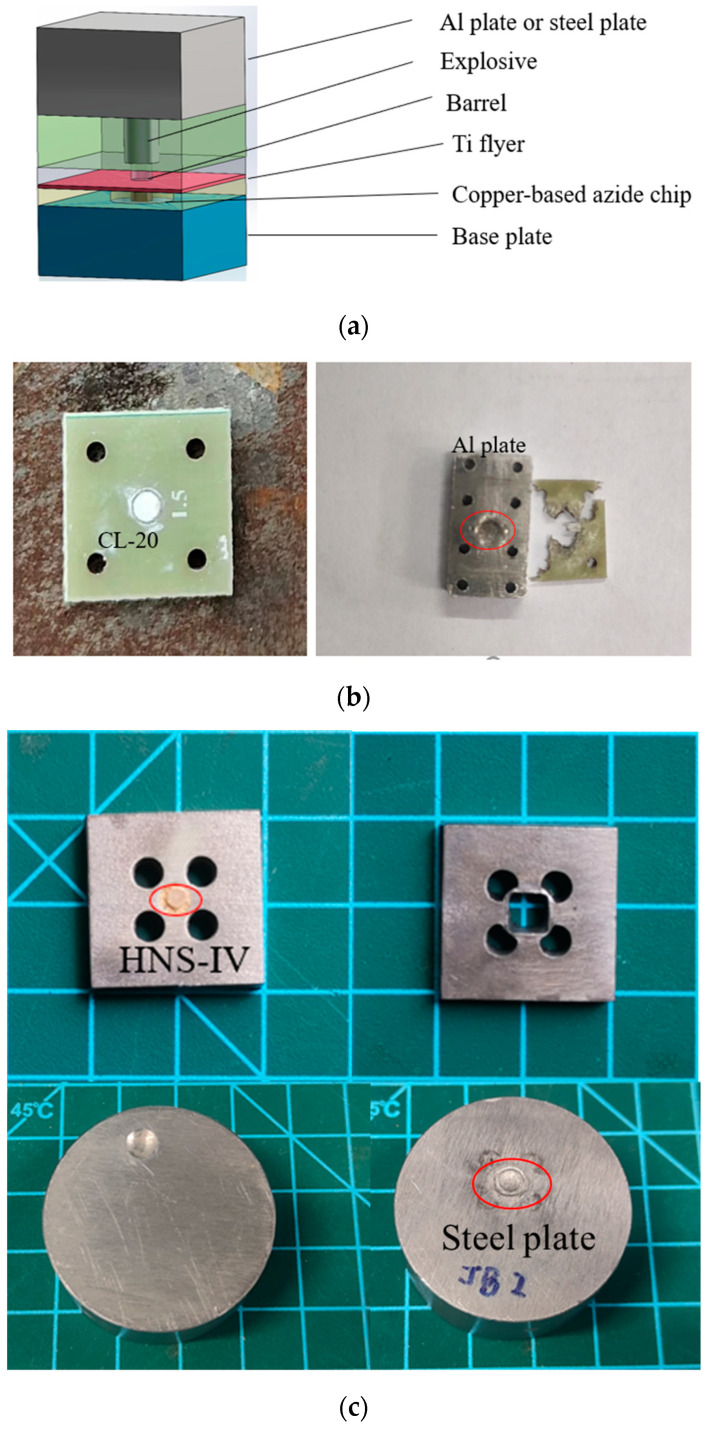
(**a**) Schematic illustration of the micro-initiator device, (**b**) initiation of CL-20 explosives and (**c**) initiation of HNS-IV explosives.

An experiment of initiating a CL-20 explosive and HNS-IV explosive using a micro-initiation device integrated with a copper-based azide chip was carried out, as shown in Figure 7. Figure 7b shows that the dent depth on the Al plate caused by the CL-20 explosive is approximately 0.72 mm, suggesting that the CL-20 explosive was successfully initiated. Figure 7c shows that the dent depth on the steel board caused by the HNS-IV explosive is approximately 0.37 mm, suggesting that the HNS-IV explosive was successfully initiated. Table 2 gives the experimental results. The diameter of the copper-based azide chip explosive was determined to be 1 mm. When the thickness of Sample a was 0.5 mm, the detonation-driven flyer failed to initiate the CL-20 and HNS-IV explosives. However, when the thickness was increased to 1 mm, the detonation-driven flyer successfully initiated both types of explosives. On the other hand, Sample b and Sample c each had a 0.5 mm thickness, and the detonation-driven flyer could successfully initiate both types of explosives.

### 3.4. Discussion

The nanocopper precursor underwent a reaction to produce Cu(N_3_)_2_ with an 8-fold volume expansion and CuN_3_ with a 4.22-fold volume expansion [21]. Previous reports have indicated that the particle size of nanoporous copper precursors prepared by the sintering method is approximately 100 nm [9]. However, after loading and molding, the expansion was constrained during the reaction process, resulting in a shallow depth of reaction, low energy of the reaction products and unstable output. In this study, the particle size of nanoporous copper was regulated by doping copper acetate using the same method, leading to the preparation of precursors with smaller particle sizes (60 nm, 30 nm). The reduced particle size resulted in a more adequate reaction, increased content of Cu(N_3_)_2_ in the product and improved output performance. Nevertheless, it is important to note that smaller precursor particle sizes do not necessarily translate to better outcomes. Decreasing the precursor particle size also reduces mechanical strength and complicates precursor molding, potentially undermining the advantages of the high energy density of copper azide. SEM results further revealed that a too-small precursor particle size (30 nm) led to the formation of raised needle-like structures on the surface of the reaction product, which was more sensitive compared to a dense and smooth surface. Taking into account output performance, safety considerations and production efficiency, we posit that a particle size of approximately 60 nm for the nanoporous copper precursor represents an optimal balance.

## 4. Conclusions

In this study, nanoporous copper with varying particle sizes was synthesized using the powder sintering method. Copper-based azide initiating charges were prepared through an in-situ azide reaction, and micro-initiators were developed to assess their performance. The results of the experiments are as follows:

(1) Doping copper acetate during the preparation of nanoporous copper via the sintered copper oxalate method was found to reduce the particle size of the nanoporous copper. By carefully controlling the addition of copper acetate, nanoporous copper particles with sizes of around 30 nm and 60 nm were successfully obtained.
(2) The gas–solid reaction principle was employed to elucidate the diverse morphologies of the reaction products derived from precursors with different particle sizes. The composition of the copper-based azide was analyzed, and the bulk energy density was calculated. A balance was achieved between precursor size, loading density and reaction depth, with the optimal particle size for the nanoporous copper precursor deemed to be 60 nm.
(3) A copper azide micro-detonator was designed to propel the flying fragments of CL-20 explosives and HNS-IV explosives for detonation testing. The experimental outcomes demonstrated that reducing the particle size of the nanoporous copper significantly enhanced the output performance. A charge thickness of 0.5 mm and a charge mass of 0.8 mg ensured stable and reliable detonation of both the CL-20 explosives and HNS-IV explosives.

In summary, this study successfully prepared nanoporous copper precursors with particle sizes of 60 nanometers and 30 nanometers while ensuring high loading density. A notable decrease in particle size compared to the data reported in reference [17] was achieved. Additionally, the copper-based azide micro-initiator designed in this study exhibited excellent initiation performance. References [22,23] reported that 6 mg and 7.5 mg of copper azide were required for initiating CL-20 explosives, respectively, while reference [24] indicated that 4.6 mg of lead azide was needed for initiating HNS-IV explosives. In contrast, the copper-based azide synthesized in this study only required 0.8 mg to reliably initiate both CL-20 and HNS-IV explosives. The copper-based azide reported in this study and the designed copper-based azide micro-initiator demonstrate significant advantages in terms of charge mass and volume, making them promising candidates for widespread application in future weapon systems.

## Figures and Tables

**Table 1:** Composition of copper-based azide.

Sample	α Cu(N_3_)_2_/%	α CuN_3_/%	α/%	Ev/J·mm^−3^
a	11.32	78.04	90.36	6.52
b	34.31	60.25	94.56	8.46
c	36.56	56.26	92.82	8.50

(a) 100 nm of NPC, (b) 60 nm of NPC and (c) 30 nm of NPC.

**Table 2:** Experiment results of initiating explosives by copper-based azide micro-initiators.

Sample	Explosive	Thickness of CA (mm)	Mass of CA (mg)	Detonation or Not
a	CL-20	0.5	0.8	no
	CL-20	1.0	1.6	yes
	HNS-IV	0.5	0.8	no
	HNS-IV	1.0	1.6	yes
b	CL-20	0.5	0.8	yes
	HNS-IV	0.5	0.8	yes
c	CL-20	0.5	0.8	yes
	HNS-IV	0.5	0.8	yes

## Data Availability

The data presented in this study are available on request from the corresponding author.
